# Recovery-based suicide prevention

**DOI:** 10.3389/fpsyt.2025.1556927

**Published:** 2025-07-23

**Authors:** Gita Ramamurthy, Robert Gregory

**Affiliations:** Department of Psychiatry, Norton School of Medicine, SUNY Upstate Medical University, Syracuse, NY, United States

**Keywords:** suicide, recovery, treatment resistance, emotion processing, psychotherapy, function, utilization, resilience

## Seriousness of suicide crisis

Suicide has become an urgent public health crisis in the United States. The age-adjusted suicide rate increased by 36% between 2000 and 2022 (1). In 2023, suicide was among the top eight leading causes of death for people ages 10–64 and the second leading cause of death for people ages 10-34 (1).

## Broadening treatment focus from the chronic illness model: restoring life meaning

The dominant treatment model in the U.S. for suicidality involves somatic treatments (primarily medication) and psychotherapy to manage psychiatric conditions and suicidality (2, 3). An underlying assumption of this model is that suicidality is generally chronic, reflecting long-standing psychiatric disorders with periodic life-threatening exacerbations. Indeed, suicidality is commonly associated with chronic mental disorders, particularly treatment-resistant depression (2, 4). Acute exacerbations of symptoms and suicidal risk are common, often requiring recurrent emergency services and/or hospitalization (5). Even after hospitalization, suicide risk remains elevated for at least 10 years (6). Since the common trajectory for suicidality involves chronicity, we call the dominant approach the “chronic illness” model.

Systematic reviews (7, 8) addressing psychotherapy for suicidality support the efficacy of psychotherapy, involving multiple approaches including cognitive-behavioral (CBT), dialectical behavioral (DBT) and psychodynamic therapies. In authoritative texts and systematic reviews, typical targets of psychotherapy involve suicidality and chronic psychiatric conditions, especially treatment-resistant depression, but do not necessarily include socio-occupational recovery (2, 3, 7–9).

Suicidality and socio-occupational dysfunction, manifested by social isolation (10) and unemployment/precarious employment (11, 12), are commonly linked, partly because both are associated with mental illness (13–18). Additionally, socio-occupational dysfunction is associated with demoralization (10, 11), a robust risk factor of suicidality (12), reflecting helplessness, hopelessness and meaninglessness (13).

Meaning, purpose and belonging are considered fundamental human needs (19, 20), integral to mental health and life satisfaction (21–24). The interpersonal theory of suicide posits that two factors underlie suicidal ideation, an intractable belief of being burdensome and thwarted belonging (16). Empirical evidence for this theory (16, 25) is reinforced by studies linking a sense of meaning/purpose and social connectedness with lower suicide risk (26–28). The US “National Strategy for Suicide Prevention” emphasizes the protective importance of socio-occupational function, given its association with a sense of meaning/purpose and belonging (29–31).

## A recovery-based model: psychotherapy targeting transdiagnostic suicide risk factors, including emotion processing deficits, to promote socio-occupational recovery

We believe resolution of chronic suicidality is possible by adding a focus on socio-occupational recovery to promote life satisfaction and social connectedness, which predict remission from suicidal ideation more robustly than psychiatric symptoms (32). Having socio-occupational recovery as the goal influences the necessary duration of psychotherapy. The recommended duration and intensity of psychotherapy for suicidality is not consensually defined. Systematic reviews reference a wide range of durations, from days to years (7–9). One systematic review reported a median duration of 12 weeks (9). However, when targeting recovery of interpersonal function, evidence suggests that a year or more is necessary (33).

Recovery of well-being and life satisfaction is facilitated by several conditions, including meeting basic needs (food, shelter and safety), developing a sense of autonomy, personal responsibility, self-acceptance and wellness skills and experiencing meaningful roles and relationships (34). The U.S. federal agency, SAMHSA (Substance Abuse and Mental Health Services Administration), emphasizes several key principles for recovery, such as hope, relationships, and person-driven holistic care, addressing biological and psychosocial vulnerabilities (35). We recommend a recovery framework to instill hope and re-engagement in life by targeting factors promoting vulnerability to socio-occupational dysfunction, treatment resistance and suicidality across diagnoses (“transdiagnostic”). These include emotion processing deficits, such as alexithymia, avoidance coping and impaired mentalization, and harsh self-criticism/lack of self- compassion (36–51).

Many of these transdiagnostic vulnerabilities can also be conceptualized as neurobiological deficits in emotion processing, the affective, cognitive and behavioral dimensions of emotion perception, interpretation and responses (52–54). Emotion processing networks include the dorsolateral prefrontal cortex, limbic cortex (medial prefrontal cortex, anterior cingulate, insula), striatum and amygdala (55). For example, alexithymia has been linked to limbic cortex dysfunction (56). Mentalization is associated medial prefrontal cortex activity (57). Notably, neurobiological changes associated with suicide appear to be independent of diagnosis, i.e., transdiagnostic (58).

Remediating emotion processing, self-compassion, and social connectedness through psychotherapy may not only reduce psychiatric symptoms, but also promote a meaningful, connected life, from functional socio-occupational recovery, to prevent suicide. Psychotherapy may reverse inflammation and many neurobiological deficits in the emotion processing system (59, 60). Additionally, psychotherapy can improve hopelessness, self-compassion, resilience, well-being and engagement in life in suicidal individuals (61–65). Preliminary evidence indicates that psychological interventions targeting alexithymia, experiential avoidance, and low self-compassion improve psychopathology, interpersonal functioning and suicide risk (66–70). Recovery of life satisfaction and sense of belonging increases well- being and predicts remission of suicidality (71).

This paper reviews evidence, including research from our institutional center, the Psychiatry High Risk Program (PHRP), supporting this treatment approach targeting transdiagnostic suicide risk factors, particularly dysfunctional emotion processing to promote recovery. Psychotherapy and adjunctive medication is given for a year, while monitoring improvement in suicidality, psychiatric symptoms and emotion processing with standardized scales (e.g., Toronto Alexithymia Scale (TAS-20) (72). This approach can lead to the resolution of suicidality, substantial recovery from psychiatric conditions and renewal of life satisfaction. The PHRP treatment model was recently designated by SAMHSA’s Suicide Prevention Resource Center as “a best practice” in suicide prevention, and received the 2023 Psychiatric Services Silver Award by the American Psychiatric Association for innovative and effective care.

## Front-loading investment in recovery to prevent suicide

We believe that the assumption of the intractability of chronic suicidality reflects insufficient outpatient treatment, particularly involving psychotherapy targeting functional recovery for at least a year.

Treatment duration and intensity can be limited by financial constraints. However, costly excessive emergency care and hospitalizations may result from inadequate outpatient treatment (73). Evidence indicates that individual psychotherapy and medication effectively reduce suicide risk and support functioning (8, 74). In adolescents, adjunctive family therapy for suicide prevention is recommended (75). This approach requires front-loading investment in recovery-focused psychiatric treatment, which we call a “recovery-based model.”

Few outpatient clinics specialize in individuals with high suicide risk. Parallels can be made with the clinical specialty clinics (CSCs) for early psychosis, promoted by NIMH and the federal government, to implement best practices (76). CSCs provide intensive, evidence-based treatment, including case management, low-dose antipsychotics, psychotherapy and occupational support. Unlike crisis-focused care, CSCs offer a long-term return in recovery/prevention of psychosis. Similarly, the approach from our institutional center, the PHRP, demonstrate effectiveness in resolving suicidality among adults and adolescents after a one-year program of psychotherapy and medication management (69, 77).

## Social determinants of suicide & deaths of despair: supporting resilience

Although psychiatric work focuses on addressing individuals, addressing a national suicide crisis requires a multi-level analysis, that includes social “determinants” (risk factors) for mental illness. Increased suicide risk from social determinants appears mediated by overwhelming stress and feelings of meaningless and disconnection.

The influential “Deaths of Despair” theory attributes the crisis of increased suicide among Americans without college degrees to financial stress and community breakdown from off-shoring/automation of manufacturing (78), leading to loss of meaning and connectedness, shame and demoralization (79). The Cultural Theory and Model of Suicide emphasizes minority stress as an important suicide risk factor (80). Minority stress is understood at two levels: The “distal” level involves experiencing chronic discrimination. Psychological consequences at the “proximal” level, such as shame from internalizing prejudicial stereotypes, or concealment, mediate negative impacts, such as suicidality and loneliness (72, 81, 82). Lower socioeconomic standing from poverty/unemployment, or ethnic/sexual minority status, are associated with shame, a lower sense of belonging and chronic, overwhelming stress (15, 83–88).

Possibly excepting ethnic minority groups, these socioeconomic characteristics are associated with increased suicide risk (87, 89).

The psychiatric consequences of marginalization and disempowerment are likely mediated by the neurotoxic and psychological effects of chronic stress (88), which can lead to demoralization (18, 90). Emotion processing deficits, exemplified by transdiagnostic suicide risk factors such as alexithymia (91), experiential avoidance (92, 93) or shame/self-blame (94, 95) can mediate and/or exacerbate the consequences of unemployment/minority stress. Psychiatrists individually cannot change social determinants, but can counteract their impact by remediating emotion processing.

## The psychiatry high risk program as an example of a recovery-based suicide prevention model

The Psychiatry High Risk Program (PHRP) offers one example of a specialized long-term outpatient recovery-based suicide prevention model. The PHRP addresses transdiagnostic vulnerabilities, such as emotion processing deficits, low self-compassion, and social alienation to promote transformational healing and resilience in suicidal youth and young adults, aged 14–40 years. The few exclusion criteria, i.e., severe intellectual impairment, severe autism, severe psychosis, and severe malnourishment, relate to suitability for full engagement in psychotherapy.

In a published study, the PHRP treatment approach led to large and significant reductions in rehospitalizations as compared to usual care, and large reductions in depression and suicide ideation, sustained for over 6 months after discharge (69). Another study demonstrated broad based improvements in highly suicidal adolescents (median 7 lifetime suicide attempts), including improvements in suicidality, depression, anxiety, self-harm, functioning, utilization, and self-compassion (77).

Initial PHRP sessions aim to inject hope by laying out a path for recovery and inviting patients to join the clinician on that path. Pros and cons of a recovery pathway versus a chronic illness pathway of care are discussed with patients, emphasizing personal choice and responsibility for health and recovery.

A core required component of the PHRP is weekly individual psychotherapy with Dynamic Deconstructive Psychotherapy (96) for up to 12 months, followed by optional monthly “maintenance” treatment. DDP has shown efficacy across a wide range of outcomes in patients with borderline personality disorder, with improvement continuing even after treatment completion (96–98). Furthermore, evidence supports that DDP promotes recovery of socio-occupational function (96, 97, 99, 100). All PHRP clinicians, including prescribers, are fully trained in DDP, enabling a common theoretical framework and minimizing potential for splitting between team members.

Individual therapy may be supplemented with medications, case management, family therapy, or group therapy within the PHRP. A team approach emphasizes a caring atmosphere and good communication among team members through weekly peer supervision of challenging cases and team meetings. Tight quality control is maintained through video recordings and quarterly outcome measures.

## Transdiagnostic vulnerabilities linked to suicidality and treatment-resistance

Although the PHRP specifically employs DDP, we believe other psychotherapies would be effective. DBT, ACT, Good Psychiatric Management and Mentalization-Based Therapy also address transdiagnostic vulnerabilities, including emotion processing deficits, linked to socio-occupational dysfunction, suicidality and treatment-resistance. For example, ACT targets avoidance coping and harsh self-criticism (101). DBT promotes emotion regulation and mindful self-compassion (102–106).

## Limitations to the recovery model

The primary difficulty in applying this model relates to front-loading costs, given insurance constraints on long-term psychotherapy. The limited supply of psychotherapists is another difficulty. Research into may clarify whether, as we believe, front-loading care over a year offers cost-effectiveness over less intensive treatment spread out over a longer term.

Furthermore, public health experts critique the neglect of population-level governmental policies, termed “universal” interventions, relative to individual-level interventions by providers (107). For example, the disproportionate increase in United States suicide rates during the 2010s, over comparable high-income countries, has been attributed to weaker social safety nets that buffer against the loss of manufacturing jobs (108). However effective a recovery approach may be, the society-wide suicide crisis is best addressed when such individual-level interventions are integrated with macroeconomic/social policies that reduce the prevalence and impact of socioeconomic risk factors for suicide (107).

## Summary

There is a need to move beyond managing chronic suicidality. We propose a Recovery-Based Model that adds functional recovery as a treatment goal. Suicidality, treatment-resistance and functional impairment commonly stem from underlying transdiagnostic biological, psychological, and socioeconomic vulnerabilities. The PHRP uses DDP for effective recovery-based suicide prevention. Other psychotherapy approaches directly targeting these vulnerabilities are likely effective also. Front-loading investment in more intensive, focused treatment may break the cycle of chronicity, reduce inpatient expenditures, resolve suicidality and improve functioning, restoring purpose and belonging.

## References

[1] National Vital Statistics System. Mortality 2018–2022. Program VSC, editor. Atlanta, GA: CDC WONDER Online Database (2024).

[2] Gold LH Frierson RL American Psychiatric Association Publishing. The American Psychiatric Association Publishing textbook of suicide risk assessment and management. 3rd ed. Washington: American Psychiatric Association Publishing (2020). p. 453. xxiii.

[3] Sudak HS. Suicide treatment 2017. In: Kaplan and sadocks comprehensive textbook of psychiatry, 10th ed. Wolters Kluwer Health, Philadelphia, PA. p. 8841–55. Kindle Edition. (2017)

[4] Kautzky A Dold M Bartova L Spies M Kranz GS Souery D. Clinical factors predicting treatment resistant depression: affirmative results from the European multicenter study. Acta Psychiatr Scand. (2019) 139:78–88. doi: 10.1111/acps.12959, PMID: 30291625 PMC6586002

[5] Fonseca Barbosa J Gama Marques J. The revolving door phenomenon in severe psychiatric disorders: A systematic review. Int J Soc Psychiatry. (2023) 69:1075–89. doi: 10.1177/00207640221143282, PMID: 37209104 PMC10338701

[6] Chung DT Ryan CJ Hadzi-Pavlovic D Singh SP Stanton C Large MM. Suicide rates after discharge from psychiatric facilities: A systematic review and meta-analysis. JAMA Psychiatry. (2017) 74:694–702. doi: 10.1001/jamapsychiatry.2017.1044, PMID: 28564699 PMC5710249

[7] Mendez-Bustos P Calati R Rubio-Ramirez F Olie E Courtet P Lopez-Castroman J. Effectiveness of psychotherapy on suicidal risk: A systematic review of observational studies. Front Psychol. (2019) 10:277. doi: 10.3389/fpsyg.2019.00277, PMID: 30837920 PMC6389707

[8] Sobanski T Josfeld S Peikert G Wagner G. Psychotherapeutic interventions for the prevention of suicide re-attempts: a systematic review. Psychol Med. (2021) 51:2525–40. doi: 10.1017/S0033291721003081, PMID: 34608856

[9] Fox KR Huang X Guzman EM Funsch KM Cha CB Ribeiro JD. Interventions for suicide and self-injury: A meta-analysis of randomized controlled trials across nearly 50 years of research. Psychol Bull. (2020) 146:1117–45. doi: 10.1037/bul0000305, PMID: 33119344

[10] Calati R Ferrari C Brittner M Oasi O Olie E Carvalho AF. Suicidal thoughts and behaviors and social isolation: A narrative review of the literature. J Affect Disord. (2019) 245:653–67. doi: 10.1016/j.jad.2018.11.022, PMID: 30445391

[11] Skinner A Osgood ND Occhipinti JA Song YJC Hickie IB. Unemployment and underemployment are causes of suicide. Sci Adv. (2023) 9:eadg3758. doi: 10.1126/sciadv.adg3758, PMID: 37436996 PMC10337900

[12] Favril L Yu R Geddes JR Fazel S. Individual-level risk factors for suicide mortality in the general population: an umbrella review. Lancet Public Health. (2023) 8:e868–e77. doi: 10.1016/S2468-2667(23)00207-4, PMID: 37898519 PMC10932753

[13] Virgolino A Costa J Santos O Pereira ME Antunes R Ambrosio S. Lost in transition: a systematic review of the association between unemployment and mental health. J Ment Health. (2022) 31:432–44. doi: 10.1080/09638237.2021.2022615, PMID: 34983292

[14] Motillon-Toudic C Walter M Seguin M Carrier JD Berrouiguet S Lemey C. Social isolation and suicide risk: Literature review and perspectives. Eur Psychiatry. (2022) 65:e65. doi: 10.1192/j.eurpsy.2022.2320, PMID: 36216777 PMC9641655

[15] Rosso BD Dekas KH Wrzesniewski A. On the meaning of work: A theoretical integration and review. Res Organizational Behav. (2010) 30:91–127. doi: 10.1016/j.riob.2010.09.001

[16] Chu C Buchman-Schmitt JM Stanley IH Hom MA Tucker RP Hagan CR. The interpersonal theory of suicide: A systematic review and meta-analysis of a decade of cross-national research. Psychol Bull. (2017) 143:1313–45. doi: 10.1037/bul0000123, PMID: 29072480 PMC5730496

[17] Costanza A Vasileios C Ambrosetti J Shah S Amerio A Aguglia A. Demoralization in suicide: A systematic review. J Psychosom Res. (2022) 157:110788. doi: 10.1016/j.jpsychores.2022.110788, PMID: 35334350

[18] Wozniewicz A Cosci F. Clinical utility of demoralization: A systematic review of the literature. Clin Psychol Rev. (2023) 99:102227. doi: 10.1016/j.cpr.2022.102227, PMID: 36462221

[19] Frankl VE. Man’s search for meaning. Boston: Beacon Press (2006). p. 165.

[20] Allen KA Gray DL Baumeister RF Leary MR. The need to belong: a deep dive into the origins, implications, and future of a foundational construct. Educ Psychol Rev. (2022) 34:1133–56. doi: 10.1007/s10648-021-09633-6, PMID: 34483627 PMC8405711

[21] Glaw X Kable A Hazelton M Inder K. Meaning in life and meaning of life in mental health care: an integrative literature review. Issues Ment Health Nurs. (2017) 38:243–52. doi: 10.1080/01612840.2016.1253804, PMID: 27929687

[22] Helliwell JF Putnam RD. The social context of well-being. Philos Trans R Soc Lond B Biol Sci. (2004) 359:1435–46. doi: 10.1098/rstb.2004.1522, PMID: 15347534 PMC1693420

[23] Harlow LL Newcomb MD. Towards a general hierarchical model of meaning and satisfaction in life. Multivariate Behav Res. (1990) 25:387–405. doi: 10.1207/s15327906mbr2503_9, PMID: 26761411

[24] Sanchez J Wadsworth JS Frain MP Umucu E Chan F. Psychiatric symptoms, psychosocial factors, and life satisfaction among persons with serious mental illness: A path analysis. J Nerv Ment Dis. (2020) 208:600–7. doi: 10.1097/NMD.0000000000001166, PMID: 32205775

[25] Silva C Smith PN Rogers M Joiner TE Foote B Van Orden KA. Clinically significant scores for thwarted belonging and perceived burden from the interpersonal needs questionnaire (INQ-15). Crisis. (2023) 44:406–14. doi: 10.1027/0227-5910/a000898, PMID: 36762737 PMC10412729

[26] Fischer IC Nichter B Feldman DB Na PJ Tsai J Harpaz-Rotem I. Purpose in life protects against the development of suicidal thoughts and behaviors in U.S. veterans without a history of suicidality: A 10-year, nationally representative, longitudinal study. J Affect Disord. (2023) 340:551–4. doi: 10.1016/j.jad.2023.08.040, PMID: 37557988

[27] Lew B Chistopolskaya K Osman A Huen JMY Abu Talib M Leung ANM. Meaning in life as a protective factor against suicidal tendencies in Chinese University students. BMC Psychiatry. (2020) 20:73. doi: 10.1186/s12888-020-02485-4, PMID: 32070298 PMC7027298

[28] Fassberg MM van Orden KA Duberstein P Erlangsen A Lapierre S Bodner E. A systematic review of social factors and suicidal behavior in older adulthood. Int J Environ Res Public Health. (2012) 9:722–45. doi: 10.3390/ijerph9030722, PMID: 22690159 PMC3367273

[29] Zechmann A Paul KI. Why do individuals suffer during unemployment? Analyzing the role of deprived psychological needs in a six-wave longitudinal study. J Occup Health Psychol. (2019) 24:641–61. doi: 10.1037/ocp0000154, PMID: 30945924

[30] Morrish N Medina-Lara A. Does unemployment lead to greater levels of loneliness? A systematic review. Soc Sci Med. (2021) 287:114339. doi: 10.1016/j.socscimed.2021.114339, PMID: 34455335 PMC8505794

[31] (OSG) OotSG. The surgeon general’s call to action to implement the national strategy for suicide prevention. Washington (DC): US Department of Health and Human Services. (2021). Available from: https://www.ncbi.nlm.nih.gov/books/NBK592704/. 37347878

[32] Teismann T Forkmann T Glaesmer H Egeri L Margraf J. Remission of suicidal thoughts: Findings from a longitudinal epidemiological study. J Affect Disord. (2016) 190:723–5. doi: 10.1016/j.jad.2015.09.066, PMID: 26600414

[33] Nordmo M Sonderland NM Havik OE Eilertsen DE Monsen JT Solbakken OA. Effectiveness of open-ended psychotherapy under clinically representative conditions. Front Psychiatry. (2020) 11:384. doi: 10.3389/fpsyt.2020.00384, PMID: 32508685 PMC7251147

[34] Dell NA Long C Mancini MA. Models of mental health recovery: An overview of systematic reviews and qualitative meta-syntheses. Psychiatr Rehabil J. (2021) 44:238–53. doi: 10.1037/prj0000444, PMID: 33734781

[35] What’s recovery? SAMHSA’s working definition. USA: Administration) SSAaMHS (2012).

[36] Hemming L Taylor P Haddock G Shaw J Pratt D. A systematic review and meta-analysis of the association between alexithymia and suicide ideation and behaviour. J Affect Disord. (2019) 254:34–48. doi: 10.1016/j.jad.2019.05.013, PMID: 31103905 PMC6599888

[37] Taylor GJ Bagby RM. New trends in alexithymia research. Psychother Psychosom. (2004) 73:68–77. doi: 10.1159/000075537, PMID: 14767148

[38] Vanheule S Desmet M Meganck R Bogaerts S. Alexithymia and interpersonal problems. J Clin Psychol. (2007) 63:109–17. doi: 10.1002/jclp.20324, PMID: 17016830

[39] Angelakis I Gooding P. Experiential avoidance in non-suicidal self-injury and suicide experiences: A systematic review and meta-analysis. Suicide Life Threat Behav. (2021) 51:978–92. doi: 10.1111/sltb.12784, PMID: 34184775

[40] Herpers PCM Neumann JEC Staal WG. Treatment refractory internalizing behaviour across disorders: an aetiological model for severe emotion dysregulation in adolescence. Child Psychiatry Hum Dev. (2021) 52:515–32. doi: 10.1007/s10578-020-01036-y, PMID: 32748274 PMC8113221

[41] Srivastava S Tamir M McGonigal KM John OP Gross JJ. The social costs of emotional suppression: a prospective study of the transition to college. J Pers Soc Psychol. (2009) 96:883–97. doi: 10.1037/a0014755, PMID: 19309209 PMC4141473

[42] Ypsilanti A. Lonely but avoidant—the unfortunate juxtaposition of loneliness and self-disgust. Palgrave Commun. (2018) 4:144. doi: 10.1057/s41599-018-0198-1

[43] Luyten P Campbell C Allison E Fonagy P. The mentalizing approach to psychopathology: state of the art and future directions. Annu Rev Clin Psychol. (2020) 16:297–325. doi: 10.1146/annurev-clinpsy-071919-015355, PMID: 32023093

[44] Hatkevich C Venta A Sharp C. Theory of mind and suicide ideation and attempt in adolescent inpatients. J Affect Disord. (2019) 256:17–25. doi: 10.1016/j.jad.2019.05.051, PMID: 31158712

[45] Hayden MC Mullauer PK Gaugeler R Senft B Andreas S. Improvements in mentalization predict improvements in interpersonal distress in patients with mental disorders. J Clin Psychol. (2018) 74:2276–86. doi: 10.1002/jclp.22673, PMID: 29998458 PMC6282818

[46] Suh H Jeong J. Association of self-compassion with suicidal thoughts and behaviors and non-suicidal self injury: A meta-analysis. Front Psychol. (2021) 12:633482. doi: 10.3389/fpsyg.2021.633482, PMID: 34122224 PMC8192964

[47] Werner AM Tibubos AN Rohrmann S Reiss N. The clinical trait self-criticism and its relation to psychopathology: A systematic review - Update. J Affect Disord. (2019) 246:530–47. doi: 10.1016/j.jad.2018.12.069, PMID: 30599378

[48] Low CA Schauenburg H Dinger U. Self-criticism and psychotherapy outcome: A systematic review and meta-analysis. Clin Psychol Rev. (2020) 75:101808. doi: 10.1016/j.cpr.2019.101808, PMID: 31864153

[49] Zuroff DC Koestner R Powers TA. Self-criticism at age 12: A longitudinal study of adjustment. Cogn Ther Res. (1994) 10:367–85. doi: 10.1007/BF02357511

[50] Michaeli Y Kalfon Hakhmigari M Dickson DJ Scharf M Shulman S. The role of change in self-criticism across young adulthood in explaining developmental outcomes and psychological wellbeing. J Pers. (2019) 87:785–98. doi: 10.1111/jopy.12433, PMID: 30260502

[51] Ypsilanti A Lazuras L Powell P Overton P. Self-disgust as a potential mechanism explaining the association between loneliness and depression. J Affect Disord. (2019) 243:108–15. doi: 10.1016/j.jad.2018.09.056, PMID: 30241025

[52] Kret ME Ploeger A. Emotion processing deficits: a liability spectrum providing insight into comorbidity of mental disorders. Neurosci Biobehav Rev. (2015) 52:153–71. doi: 10.1016/j.neubiorev.2015.02.011, PMID: 25725415

[53] Angus LE Paivio SC. Narrative processes in emotion-focused therapy for trauma. Washington DC: American Psychological Association (2017) p. 3–7.

[54] Milan S Carlone C Printz D Perez SD. Understanding children’s emotions: differences in mothers with a history of childhood maltreatment. Child Maltreat. (2022) 27:33–42. doi: 10.1177/1077559520972188, PMID: 33176473

[55] Mann JJ Rizk MM. A brain-centric model of suicidal behavior. Am J Psychiatry. (2020) 177:902–16. doi: 10.1176/appi.ajp.2020.20081224, PMID: 32998550 PMC7676389

[56] van der Velde J Servaas MN Goerlich KS Bruggeman R Horton P Costafreda SG. Neural correlates of alexithymia: a meta-analysis of emotion processing studies. Neurosci Biobehav Rev. (2013) 37:1774–85. doi: 10.1016/j.neubiorev.2013.07.008, PMID: 23886515

[57] Arioli M Cattaneo Z Ricciardi E Canessa N. Overlapping and specific neural correlates for empathizing, affective mentalizing, and cognitive mentalizing: A coordinate-based meta-analytic study. Hum Brain Mapp. (2021) 42:4777–804. doi: 10.1002/hbm.25570, PMID: 34322943 PMC8410528

[58] Fan S Lippard ETC Sankar A Wallace A Johnston JAY Wang F. Gray and white matter differences in adolescents and young adults with prior suicide attempts across bipolar and major depressive disorders. J Affect Disord. (2019) 245:1089–97. doi: 10.1016/j.jad.2018.11.095, PMID: 30699851 PMC6903411

[59] Shields GS Spahr CM Slavich GM. Psychosocial interventions and immune system function: A systematic review and meta-analysis of randomized clinical trials. JAMA Psychiatry. (2020) 77:1031–43. doi: 10.1001/jamapsychiatry.2020.0431, PMID: 32492090 PMC7272116

[60] Marwood L Wise T Perkins AM Cleare AJ. Meta-analyses of the neural mechanisms and predictors of response to psychotherapy in depression and anxiety. Neurosci Biobehav Rev. (2018) 95:61–72. doi: 10.1016/j.neubiorev.2018.09.022, PMID: 30278195 PMC6267850

[61] Hawton K Witt KG Salisbury TLT Arensman E Gunnell D Hazell P. Psychosocial interventions following self-harm in adults: a systematic review and meta-analysis. Lancet Psychiatry. (2016) 3:740–50. doi: 10.1016/S2215-0366(16)30070-0, PMID: 27422028

[62] Ferrari M Hunt C Harrysunker A Abbott MJ Beath AP Einstein DA. Self-compassion interventions and psychosocial outcomes: a meta-analysis of RCTs. Mindfulness Mindfulness. (2019) 10:1455–73. doi: 10.1007/s12671-019-01134-6

[63] Ferrari M Hunt C Harrysunker A Abbott MJ Beath AP Einstein DA. Road to resilience: a systematic review and meta-analysis of resilience training programmes and interventions. BMJ Open. (2018) 8:e017858. doi: 10.1136/bmjopen-2017-017858, PMID: 29903782 PMC6009510

[64] Briggs S Netuveli G Gould N Gkaravella A Gluckman NS Kangogyere P. The effectiveness of psychoanalytic/psychodynamic psychotherapy for reducing suicide attempts and self-harm: systematic review and meta-analysis. Br J Psychiatry. (2019) 214:320–8. doi: 10.1192/bjp.2019.33, PMID: 30816079

[65] Ducasse D Jaussent I Arpon-Brand V Vienot M Laglaoui C Beziat S. Acceptance and commitment therapy for the management of suicidal patients: A randomized controlled trial. Psychother Psychosom. (2018) 87:211–22. doi: 10.1159/000488715, PMID: 29874680

[66] Johnson SB Goodnight BL Zhang H Daboin I Patterson B Kaslow NJ. Compassion-based meditation in african americans: self-criticism mediates changes in depression. Suicide Life Threat Behav. (2018) 48:160–8. doi: 10.1111/sltb.12347, PMID: 28326598

[67] Ogrodniczuk JS Sochting I Piper WE Joyce AS. A naturalistic study of alexithymia among psychiatric outpatients treated in an integrated group therapy program. Psychol Psychother. (2012) 85:278–91. doi: 10.1111/j.2044-8341.2011.02032.x, PMID: 22903919

[68] Shorey RC Gawrysiak MJ Elmquist J Brem M Anderson S Stuart GL. Experiential avoidance, distress tolerance, and substance use cravings among adults in residential treatment for substance use disorders. J Addict Dis. (2017) 36:151–7. doi: 10.1080/10550887.2017.1302661, PMID: 28358236 PMC6126664

[69] Thomas JG Sperry SD Shields RJ Gregory RJ. A novel recovery-based suicide prevention program in upstate new york. J Addict Dis. (2022) 73:701–4. doi: 10.1176/appi.ps.202100162. pidpmid., PMID: 34704773

[70] Yela JR Crego A Buz J Sanchez-Zaballos E Gomez-Martinez MA. Reductions in experiential avoidance explain changes in anxiety, depression and well-being after a mindfulness and self-compassion (MSC) training. Psychol Psychother. (2022) 95:402–22. doi: 10.1111/papt.12375, PMID: 34904363

[71] Koivumaa-Honkanen H Honkanen R Viinamaki H Heikkila K Kaprio J Koskenvuo M. Life satisfaction and suicide: a 20-year follow-up study. Am J Psychiatry. (2001) 158:433–9. doi: 10.1176/appi.ajp.158.3.433, PMID: 11229985

[72] Meyer IH. Prejudice, social stress, and mental health in lesbian, gay, and bisexual populations: conceptual issues and research evidence. Psychol Bull. (2003) 129:674–97. doi: 10.1037/0033-2909.129.5.674, PMID: 12956539 PMC2072932

[73] Casey M Perera D Enticott J Vo H Cubra S Gravell A. High utilisers of emergency departments: the profile and journey of patients with mental health issues. Int J Psychiatry Clin Pract. (2021) 25:316–24. doi: 10.1080/13651501.2021.1904998, PMID: 33945750

[74] Mann JJ Michel CA Auerbach RP. Improving suicide prevention through evidence-based strategies: A systematic review. Am J Psychiatry. (2021) 178:611–24. doi: 10.1176/appi.ajp.2020.20060864, PMID: 33596680 PMC9092896

[75] Frey LM Hunt QA Russon JM Diamond G. Review of family-based treatments from 2010 to 2019 for suicidal ideation and behavior. J Marital Fam Ther. (2022) 48:154–77. doi: 10.1111/jmft.12568, PMID: 34710242

[76] George P Ghose SS Goldman HH O’Brien J Daley TC Dixon LB. Growth of coordinated specialty care in the United States with changes in federal funding policies: 2014-2018. Psychiatr Serv. (2022) 73(12):1346–51. doi: 10.1176/appi.ps.202100600. Epub 2022 Jun 16. Erratum in: Psychiatr Serv. (2022) 73(12):1189. doi: 10.1176/appi.ps.202100600correction, PMID: 35707858 PMC9722492

[77] Shields RJ Helfrich JP Gregory RJ. Dynamic deconstructive psychotherapy for suicidal adolescents: effectiveness of routine care in an outpatient clinic. Int J Environ Res Public Health. (2024) 21. doi: 10.3390/ijerph21070929, PMID: 39063505 PMC11276631

[78] Case A Deaton A. The great divide: education, despair, and death. Annu Rev Econom. (2022) 14:1–21. doi: 10.1146/annurev-economics-051520-015607, PMID: 35990244 PMC9389919

[79] Friedman SR Krawczyk N Perlman DC Mateu-Gelabert P Ompad DC Hamilton L. The opioid/overdose crisis as a dialectics of pain, despair, and one-sided struggle. Front Public Health. (2020) 8:540423. doi: 10.3389/fpubh.2020.540423, PMID: 33251171 PMC7676222

[80] Chu JP Goldblum P Floyd R Bongar B. The cultural theory and model of suicide. Appl Prev Psychol. (2010) 14:25–40. doi: 10.1016/j.appsy.2011.11.001

[81] Frost DM Meyer IH. Minority stress theory: Application, critique, and continued relevance. Curr Opin Psychol. (2023) 51:101579. doi: 10.1016/j.copsyc.2023.101579, PMID: 37270877 PMC10712335

[82] Huang YT Chan RCH. Effects of sexual orientation concealment on well-being among sexual minorities: How and when does concealment hurt? J Couns Psychol. (2022) 69:630–41. doi: 10.1037/cou0000623, PMID: 35696152

[83] Cenat JM. Complex racial trauma: evidence, theory, assessment, and treatment. Perspect Psychol Sci. (2023) 18:675–87. doi: 10.1177/17456916221120428, PMID: 36288462 PMC10186562

[84] Eales MJ. Shame among unemployed men. Soc Sci Med. (1989) 28:783–9. doi: 10.1016/0277-9536(89)90107-x, PMID: 2705012

[85] Stewart MJ Makwarimba E Reutter LI Veenstra G Raphae D Love R. Poverty, sense of belonging and experiences of social isolation. J Poverty. (2009) 13:173–95. doi: 10.1080/10875540902841762

[86] Wu Z Finnsdottir M. Perceived racial and cultural discrimination and sense of belonging in Canadian society. Can Rev Sociol. (2021) 58:229–49. doi: 10.1111/cars.12339, PMID: 33913252

[87] Garcia J Vargas N Clark JL Magana Alvarez M Nelons DA Parker RG. Social isolation and connectedness as determinants of well-being: Global evidence mapping focused on LGBTQ youth. Glob Public Health. (2020) 15:497–519. doi: 10.1080/17441692.2019.1682028, PMID: 31658001 PMC7093214

[88] Sederer LI. The social determinants of mental health. Glob Public Health (2016) 67:234–5. doi: 10.1176/appi.ps.201500232, PMID: 26522677

[89] Liu S Morin SB Bourand NM DeClue IL Delgado GE Fan J. Social vulnerability and risk of suicide in US adults, 2016-2020. JAMA Netw Open. (2023) 6:e239995. doi: 10.1001/jamanetworkopen.2023.9995, PMID: 37099296 PMC10134005

[90] Briggs L. Is the degree of demoralization found among refugee and migrant populations a social-political problem or a psychological one? Eur J Psychiatry. (2013) 27:27–35. doi: 10.4321/S0213-61632013000100004

[91] Denning DM Brown TA. Examining the prospective effects of alexithymia and minority stress on emotion regulation in sexual minority adults. Affect Sci. (2025). doi: 10.1007/s42761-024-00290-w, PMID: 40605937 PMC12209481

[92] Mann AM Naugle AE Lieberman E. Experiential avoidance and emotion dysregulation as mediators in the LGBTQ minority stress model. Arch Sex Behav. (2022) 51:3443–56. doi: 10.1007/s10508-022-02376-7, PMID: 35951198

[93] Mantler J Matejicek A Matheson K Anisman H. Coping with employment uncertainty: a comparison of employed and unemployed workers. J Occup Health Psychol. (2005) 10:200–9. doi: 10.1037/1076-8998.10.3.200, PMID: 16060724

[94] Barrita AM Wong-Padoongpatt G. Ethnic identity and resilience: a moderated mediation analysis of protective factors for self-blame and racial microaggressions. Front Psychol. (2023) 14:1198375. doi: 10.3389/fpsyg.2023.1198375, PMID: 37457064 PMC10343435

[95] Extremera N Rey L. Health-related quality of life and cognitive emotion regulation strategies in the unemployed: a cross-sectional survey. Health Qual Life Outcomes. (2014) 12:172. doi: 10.1186/s12955-014-0172-6, PMID: 25432102 PMC4263041

[96] Gregory RJ Chlebowski S Kang D Remen AL Soderberg MG Stepkovitch J. A controlled trial of psychodynamic psychotherapy for co-occurring borderline personality disorder and alcohol use disorder. Psychother (Chic). (2008) 45:28–41. doi: 10.1037/0033-3204.45.1.28, PMID: 22122363

[97] Gregory RJ Sachdeva S. Naturalistic outcomes of evidence-based therapies for borderline personality disorder at a medical university clinic. Am J Psychother. (2016) 70:167–84. doi: 10.1176/appi.psychotherapy.2016.70.2.167, PMID: 27329405

[98] Majdara E Rahimmian I Talepassand S Gregory RJ. A randomized trial of dynamic deconstructive psychotherapy in Iran for borderline personality disorder. J Am Psychoanal Assoc. (2019) 67:NP1–7. doi: 10.1177/0003065119891390, PMID: 31850790

[99] Gregory RJ. Borderline attributions. Am J Psychother. (2007) 61:131–47. doi: 10.1176/appi.psychotherapy.2007.61.2.131, PMID: 17760318

[100] Storebo OJ Stoffers-Winterling JM Vollm BA Kongerslev MT Mattivi JT Jorgensen MS. Psychological therapies for people with borderline personality disorder. Cochrane Database Syst Rev. (2020) 5:CD012955. doi: 10.1002/14651858.CD012955.pub2, PMID: 32368793 PMC7199382

[101] Fawson S Moon Z Novogrudsky K Moxham F Forster K Tribe I. Acceptance and commitment therapy processes and their association with distress in cancer: a systematic review and meta-analysis. Health Psychol Rev. (2024) 18:456–77. doi: 10.1080/17437199.2023.2261518, PMID: 37746724 PMC11332408

[102] McMain S Korman LM Dimeff L. Dialectical behavior therapy and the treatment of emotion dysregulation. J Clin Psychol. (2001) 57:183–96. doi: 10.1002/1097-4679(200102)57:2<183::aid-jclp5>3.0.co;2-y 11180146

[103] Linehan M. DBT skills training handouts and worksheets. 2nd ed. New York: The Guilford Press (2015). p. 422.

[104] Patrichi A Rimbu R Miu AC Szentagotai-Tatar A. Loneliness and emotion regulation: A meta-analytic review. Emotion. (2025) 25:755–74. doi: 10.1037/emo0001438, PMID: 39541515

[105] Fornaro M Caiazza C Pistone L Crincoli W Pezone R De Prisco M. Atypical depression and emotion dysregulation: Clinical and psychopathological features. J Affect Disord. (2025) 376:410–21. doi: 10.1016/j.jad.2025.02.034, PMID: 39965674

[106] Colmenero-Navarrete L Garcia-Sancho E Salguero JM. Relationship between emotion regulation and suicide ideation and attempt in adults and adolescents: A systematic review. Arch Suicide Res. (2022) 26:1702–35. doi: 10.1080/13811118.2021.1999872, PMID: 34821201

[107] Pirkis J Dandona R Silverman M Khan M Hawton K. Preventing suicide: a public health approach to a global problem. Lancet Public Health. (2024) 9:e787–e95. doi: 10.1016/S2468-2667(24)00149-X, PMID: 39265611

[108] Case A Deaton A. Mortality and morbidity in the 21(st) century. Brookings Pap Econ Act. (2017) 2017:397–476. doi: 10.1353/eca.2017.0005, PMID: 29033460 PMC5640267

